# Roles of Virtual Screening and Molecular Dynamics Simulations in Discovering and Understanding Antimalarial Drugs

**DOI:** 10.3390/ijms24119289

**Published:** 2023-05-26

**Authors:** Searle S. Duay, Rianne Casey Y. Yap, Arturo L. Gaitano, June Alexis A. Santos, Stephani Joy Y. Macalino

**Affiliations:** 1 Department of Chemistry, De La Salle University, Manila 0922, Philippines; rianne_yap@dlsu.edu.ph; 2 Chemistry Department, Adamson University, Manila 1000, Philippines; arturo.gaitano@adamson.edu.ph (A.L.G.III); june.alexis.santos@adamson.edu.ph (J.A.A.S.)

**Keywords:** malaria, in silico, virtual screening, machine learning, docking, molecular dynamics

## Abstract

Malaria continues to be a global health threat, with approximately 247 million cases worldwide. Despite therapeutic interventions being available, patient compliance is a problem due to the length of treatment. Moreover, drug-resistant strains have emerged over the years, necessitating urgent identification of novel and more potent treatments. Given that traditional drug discovery often requires a great deal of time and resources, most drug discovery efforts now use computational methods. In silico techniques such as quantitative structure-activity relationship (QSAR), docking, and molecular dynamics (MD) can be used to study protein-ligand interactions and determine the potency and safety profile of a set of candidate compounds to help prioritize those tested using assays and animal models. This paper provides an overview of antimalarial drug discovery and the application of computational methods in identifying candidate inhibitors and elucidating their potential mechanisms of action. We conclude with the continued challenges and future perspectives in the field of antimalarial drug discovery.

## 1. Introduction

Malaria is an infectious disease caused by protozoan parasites belonging to the Plasmodium genus, with about 247 million cases globally across 84 malaria-endemic countries, including the Philippines [1]. These parasites are transmitted to humans through the bites of infected female Anopheles mosquitoes, which inject sporozoites, the infectious form of the parasite, into the host’s bloodstream [2,3]. Once inside the human body, sporozoites rapidly migrate to the liver, infecting hepatocytes, and begin asymptomatic asexual reproduction. Subsequently, the parasites emerge into merozoites, which are released into the bloodstream and invade red blood cells (RBCs). Within the RBCs, the parasites multiply, destroying the cells and releasing more merozoites, which continue to infect other RBCs [2].

During the infection, *Plasmodium* undergoes multiple stages in its life cycle, including the blood-stage in which the disease starts to manifest in humans. At this stage, the parasite degrades the host hemoglobin for survival [4]. One of the products, Fe(III)-protoporphyrin IX (Fe(III)PPIX), accumulates and causes lipid peroxidation and membrane disruption [5]. Experimental studies have been published on the mechanism of the disease [6], the development of small-molecule therapeutic drugs [7], and the elucidation of the mechanism of action of these drugs [7,8]. Due to rapid technological advancements, computational studies became highly significant in further understanding the mechanisms of malarial infection and developing anti-malarial drugs. The review paper of Muller and Hyde [8] covers the modes of action and resistance mechanisms of antimalarial drugs, but the role of computational studies was not highlighted. This paper will review literature that utilized structure- and ligand-based drug discovery methods, as well as molecular dynamics simulations, over the past years that contributed to the research on malaria. This review will discuss recent computational studies, and thus, it is important to note that the inhibitors involved are all in the pre-clinical stage of the drug discovery pipeline. None of the inhibitors have undergone therapeutic evaluation and we do not intend to suggest any prioritization of these molecules.

## 2. Current Treatment Strategies and Clinical Candidates Targeting Malaria

While the incidence of malaria has notably decreased in the last two decades, a slight increase was observed in 2020, potentially in line with the interruption of several health and medical services as COVID-19 was prioritized. WHO aims to decrease malaria’s global incidence and mortality by at least 90% in the next seven years. To achieve this goal, reliable diagnosis, surveillance, and the successful application of current interventions are needed. Existing therapeutic strategies targeting malaria include artemisinin-based combination therapy (ACT), which often requires 3 days of treatment, leading to poor patient compliance. Moreover, increasing artemisinin resistance due to target mutations has become a concern, indicating the need for more research to find better and novel therapeutics to combat this disease. Fortunately, several non-artemisinin compounds, such as tetrahydro-β-carboline derivatives, mefloquine, and piperaquine (discussed later in the text), are currently being evaluated for their efficacy and safety.

**Figure 1:**
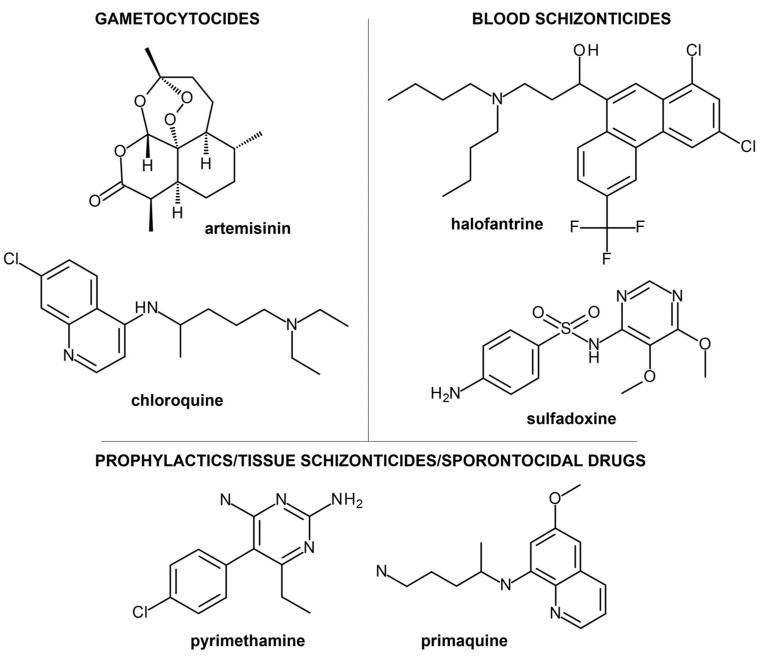
Some known antimalarial drugs separated into classes: gametocytocides, blood schizonticides, tissue schizonticides, prophylactics, and sporontocides.

Antimalarial drugs can be divided into five classes: gametocides, blood schizonticides, tissue schizonticides, sporontocides, and prophylactics (Figure 1) [9]. Gametocides can destroy the parasites’ gametocytes in the blood stage of the malaria life cycle, resulting in the inhibition of malaria transfer from an infected individual to an uninfected *Anopheles* mosquito. This is the primary mechanism of artemisinin and chloroquine. Antimalarial drugs such as pyrimethamine and primaquine exhibit several modes of action. These compounds work as prophylactics, often given to prevent malarial infections in individuals with weak immune functions. They also function as tissue schizonticides, which inhibit infection relapse due to *Plasmodium ovale* and *Plasmodium vivax* dormant forms or hypnozoites in the liver stage of the malaria life cycle, and as sporontocides, which hinder the development of oocytes in the mosquito stage, leading to inhibition of disease transmission.

Current antimalarial drug discovery research focuses on two primary goals: (1) candidates that can target resistant strains and (2) highly potent candidates that can be administered in shorter treatment regimens. One of the solutions identified for this is using partner drugs wherein artemisinin or a derivative is partnered with another drug to produce the intended therapeutic effect. However, further research on shortening or minimizing the treatment to a single-dose cure is still needed. Additionally, candidates that can target both the asexual and sexual stages, along with the hypnozoites of *P. vivax* and *P. ovale,* are of particular interest, given that hypnozoites can potentially lead to multiple malaria episodes in just a single infection.

**Figure 2:**
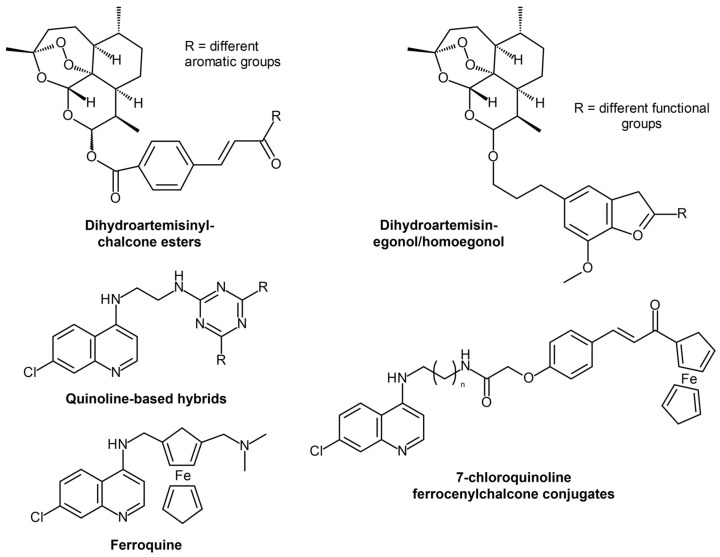
Artemisinin and non-artemisinin hybrid candidate compounds. Redrawn and reproduced with permission from Elsevier [10,11,12,16]. Further permissions related to the material excerpted should be directed to the primary source.

Artemisinin-hybridized compounds (Figure 2) have been tested against malaria in the last several years to answer artemisinin resistance. One example is dihydroartemisinyl-chalcone esters, which were found to be potent against chloroquinine-resistant and -sensitive strains and are thermally stable, which means they can easily be stored in high-temperature facilities in tropical regions where malaria is often endemic [10]. Another set of interesting antimalarial candidates includes hybrids of artemisinin and other natural products, such as homoegonol and thymoquinone [11], which had high antiplasmodial efficacy and potency, showing better activity than chloroquine. Non-artemisinin-hybridized compounds (Figure 2) based on quinoline [12,13,14] showed inhibition against hemozoin formation and the importance of an aromatic lipophilic side chain to the inhibitory activity. Additionally, ferrocene-based hybrids [15,16,17,18] have also been tested, with ferroquine exhibiting the most potential as it was found to be safe and effective against resistant strains and has a high half-life while being well-tolerated even at high repeated dosage [18].

## 3. Computer-Aided Anti-Malarial Drug Discovery

### 3.1. Structure-Based Methods

The availability of three-dimensional (3D) protein structural information has facilitated drug discovery and development for numerous diseases, as the binding site information obtained from a drug target can guide the design of potent small molecule candidates. Common structure-based methods include homology modeling, molecular docking, receptor-based pharmacophore modeling and screening, and molecular dynamics simulation, most of which have been applied for anti-malarial drug discovery in recent years (Table 1). These methods have been extremely helpful in facilitating antimalarial drug discovery, especially with the advent of artemisinin- and piperaquine-resistance, along with decreased efficacy of artemisinin partner drugs in ACT. With the help of rational design, compounds with novel structures and mechanisms, better pharmacokinetic properties and safety profiles, and better potency against resistant strains can be designed and developed.

Highlighted in this article are a number of studies that took advantage of existing software and protein and ligand information to identify antimalarial candidates.

#### 3.1.1. Reverse Docking of β-Carboline Derivatives

**Figure 3:**
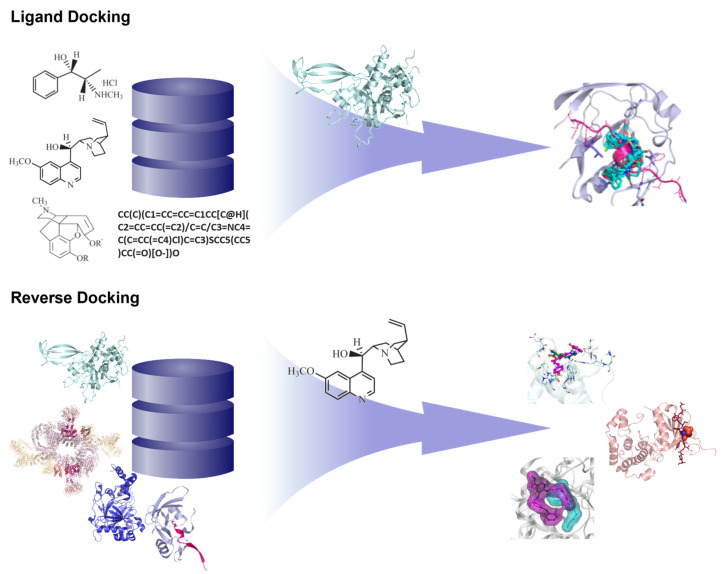
Traditional ligand docking vs. Reverse docking. Traditional ligand docking employs compound databases and screens ligand structures against one target protein, whereas reverse docking makes use of protein databases which are screened against a compound with desirable drug properties.

Most structure-based virtual screening focuses on using a ligand library to identify potential hits that can inhibit or activate a given target. However, virtual screening can also be performed using a library of proteins to identify potential drug target/s of a given compound in a process known as inverse or reverse docking (Figure 3) [32].

β-Carboline is a notable scaffold found in several natural products and currently marketed drugs. Several β-Carboline compounds have also displayed potent antimalarial activities [33]. A study tested the antimalarial activity of tetrahydro-β-carboline (THβC) derivatives against chloroquine-sensitive and -resistant strains of *P. falciparum* [23]. Based on the in vitro results, the derivatives showed better potency when the pyridine ring was present at the C1 position of the compound. The introduction of the 1,3-dioxolane ring also led to better potency, along with the manipulation of stereochemistry, wherein trans-diasteromers showed better overall activity. From the series of testing and optimization, compounds **11a**, **9a**, and **9b** were identified as the best compounds against both the sensitive and resistant strains of *P. falciparum*. Cell toxicity assays were also performed to determine the safety of the identified leads. Compound **9a** was found to be toxic against Hela cells but nontoxic towards dermal fibroblasts (normal cells), suggesting that it has a desirable safety profile for human consumption. To further validate the activity and safety of the lead, in vivo testing against *P. berghei* was also explored. Compound **9a** displayed LD50 > 4 g/kg, indicating that the compound is safe for oral administration at high dosages. As a monotherapy, it exhibited an effective dose of 27.74 mg/kg with a high potential to inhibit the development of the parasite and improve the chances of survival of the treated animals. Combined with a high dose of artesunate, a chemosupression of 99.69% was observed on day 5, with complete parasitic clearance seen in the treated mice after 28 days.

**Figure 4:**
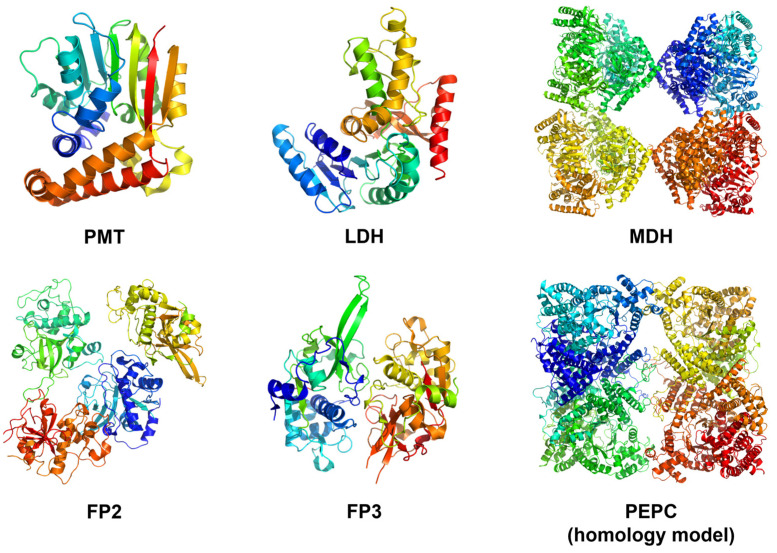
Reverse docking targets for β-Carboline derivatives. Protein structures are shown in cartoon representations with the backbone carbons colored in rainbow spectrum.

Several studies have established the roles of phosphoenolpyruvate carboxylase (PEPC), phosphoethanolamine methyltransferase (PMT), lactate dehydrogenase (LDH), malate dehydrogenase (MDH), falcipain-2 (FP2), and falcipain-3 (FP3) in malarial pathogenesis. The β-Carboline derivatives, along with artesunate, were docked against these proteins to study their potential binding interactions and predict their specific targets. Prior to molecular docking, the protein structures were prepared (Figure 4). The PDB structures of the PMT (PDB ID: 3UJ9), LDH (PDB ID: 1LDG), MDH (PDB ID: 5NFR), FP2 (PDB ID: 3BPF), and FP3 (PDB ID: 3BPM) were prepared by removing water molecules and co-crystal ligands, fixing protonation, and modeling missing residues and loops. At the same time, homology modeling of the PEPC receptor had to be performed using the UniProt sequence Q8ILJ7.

While the 3D target structures solved from experimental techniques such as NMR, X-ray crystallography, and Cryo-EM can often provide vast information that can be used for drug design and development, thousands of potential drug targets are still without experimentally elucidated structures. AlphaFold and other Artificial Intelligence (AI)-based techniques in structural biology are also still in the stage of being established. Despite the existence of all these techniques, homology or comparative modeling remains an attractive alternative when there is no 3D structural data on hand for a drug target of interest. Homology modeling takes advantage of the assumption that structures with similar sequences will have similar structures and functions. Therefore, a template with a known 3D structure can be used to model any target with a similar amino acid sequence [34], upon which further computational experiments can be performed, such as molecular docking or dynamics.

**Figure 5:**
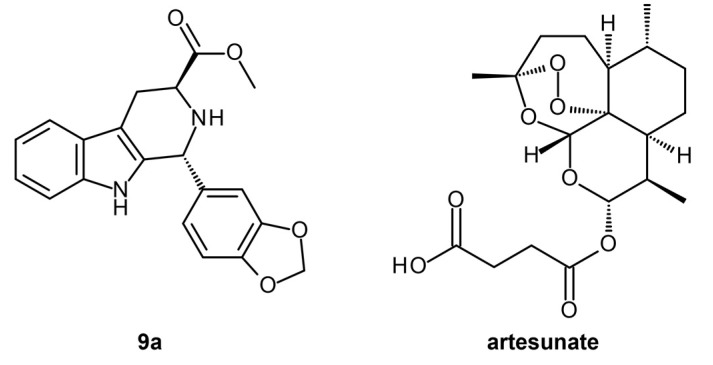
Compound **9a** and artesunate structures. Compound **9a** is redrawn and reproduced with permission from ACS Omega [23]. Further permissions related to the material excerpted should be directed to the primary source.

VLifeMDS 4.6 Bio Predicta module [35] was employed for the reverse docking. Consistent with the in vitro and in vivo results, compound **9a** showed an excellent docking profile against three target proteins, FP3 (−34.27 kcal/mol), PEPC (−37.44 kcal/mol), and LDH (−59.14 kcal/mol), compared to artesunate (−8.64, −8.24, and −51.92 kcal/mol, respectively). Several hydrogen bonds, hydrophobic, and van der Waals interactions were observed for compound **9a** and the protein targets, accounting for the good binding energies. Artesunate exhibited desirable binding with MDH and FP2 with energies of −54.62 kcal/mol and −62.45 kcal/mol, respectively, compared to **9a**, which displayed −17.24 kcal/mol and −60.32 kcal/mol, respectively, for the targets. This suggests that if used as a combination therapy, **9a** and artesunate (Figure 5) can target different proteins to hinder malarial pathogenesis [23].

#### 3.1.2. *Anopheles gambiae* Trehalase Ab Initio Modeling and Hit Identification

One of the targets identified for malaria drug design is trehalase, an enzyme responsible for reducing trehalose, resulting in two glucose molecules. In malaria, *Anopheles gambiae* carries the pathogen *P. falciparum*, which accounts for most malignant cases of malaria in sub-Saharan Africa. The pathogen’s gametocytes source trehalose to protect against desiccation and heat, stabilize membranes and proteins, and encourage reproduction by at least a thousandth-fold [36]. Moreover, trehalase has become an attractive target for malaria vectors due to its specificity towards insects, deeming it safe for human consumption [20].

A recent study used ab initio modeling, molecular docking, and molecular dynamics simulations to identify potential inhibitors for *Anopheles gambiae* trehalase (*Ag*Tre) [37]. In theory, the structure of a protein and the way it will fold is encoded into its sequence. This is the fundamental concept being followed by ab initio modeling, wherein a protein structure is generated with only the amino acid sequence of the target protein [38]. Adedeji et al. generated an ab initio 3D structure of *Ag*Tre through three web servers, namely, RoseTTAFold [39,40], C-I-TASSER [41], and I-TASSER [42]. The RoseTTAFold server utilizes deep learning-based modeling software that considers protein sequences, possible protein 3D conformations, and protein interaction [40] and provided an *Ag*Tre model that had the best confidence score, QMEAN score, and Ramachandran favorability (0.85, −0.10, 98.06%, respectively). Hence, the RoseTTAFold-based model was used for the subsequent virtual procedures.

Active site prediction was also performed through the CASTp [43,44] and PrankWeb [45,46] servers. Often, the binding pocket of a target is identified through the presence of a co-crystal ligand or a deep pocket with key residues that were established through mutation studies. However, this is not always the case, especially for proteins with no experimentally validated 3D structures. In these cases, additional analyses in the form of binding site prediction are needed to continue with structure-based methods such as docking or receptor-based pharmacophore modeling. CASTp determines and measures potential binding pockets in a query protein structure based on annotations from Protein Data Bank (PDB), Swiss-Prot, and Online Mendelian Inheritance in Man (OMIM) [44]. PrankWeb, on the other hand, makes use of machine learning (ML) and evolutionary sequence conservation information to predict the properties of a potential binding site and assess its ligandability [45]. Overlapping ideal pocket scores and binding probabilities of active site residues from both servers were used to define the active site and, thus, the grid box.

**Figure 6:**
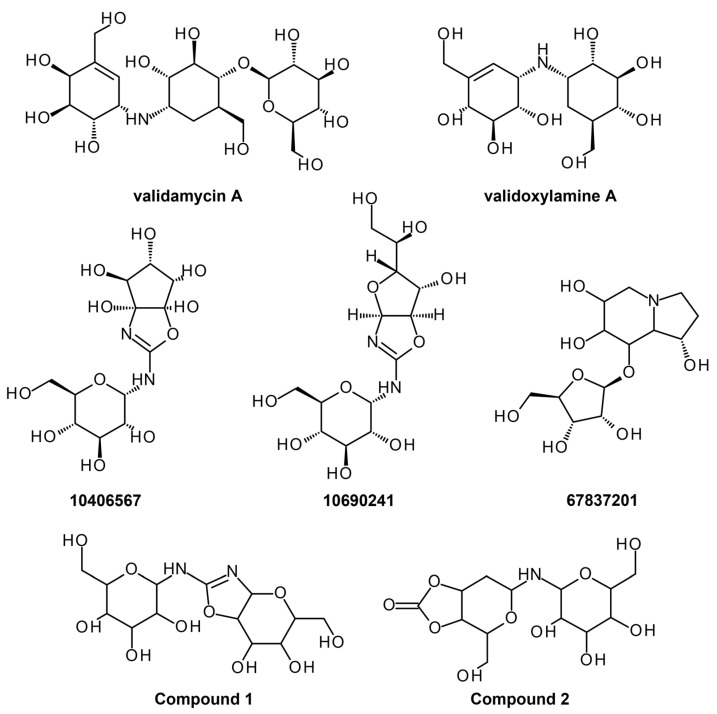
Validamycin A and its analogues.

For the virtual screening, validamycin A, a commercially available compound that exhibits trehalase inhibition for different species, was used as a control to search for potential hits in the PubChem database. Molecular docking was performed via AutoDock Vina [47] in PyRx [48], wherein validamycin A and other trehalase inhibitors were docked to predict their binding affinities and interactions with the trehalase model. Molecular docking is one of the oldest and most well-known structure-based methods that can be used to predict the binding orientation of a ligand, as well as its interactions and affinity with a given target [49]. Validamycin A (Figure 6) displayed an energy of −5.4 kcal/mol, which is higher than most previously reported trehalase inhibitors, except for validoxylamine A (Figure 6), which showed a calculated binding energy of −8.8 kcal/mol. It was discovered that validamycin A showed poor binding to *Ag*Tre due to the presence of only a single van der Waals interaction with active site residue Tyr507. Furthermore, the bulk of its binding site interactions is composed of hydrogen bonds with non-active site residues. Validoxylamine A, on the other hand, had several van der Waals interactions with active site residues and was used to identify the top nine ligands with similar or better binding energy to trehalase (<−8.8 kcal/mol). Additionally, the ligands were screened using Pan-Assay INterference compoundS (PAINS) [50,51], a set of substructural features that can be employed to determine if ligand hits are promiscuous compounds or non-specific binders. Based on the lack of PAINS alert, the identified ligands in this study are proposed to be non-promiscuous and target-specific. Amongst the top nine hit compounds, **10406567**, **10690241**, and **67837201** (C_13_H_22_N_2_O_10_; C_13_H_22_N_2_O_10_; C_13_H_23_NO_8_; Figure 6) were predicted to be non-permeable to the blood-brain barrier, and non-toxic accounted for by its respiratory toxicity, drug-induced liver toxicity percentage, and its metabolically stable structure. As such, these compounds were further optimized to supply 120 inhibitory compounds, wherein Compound **1** (2-[6,7-dihydroxy-5-(hydroxymethyl)-3aH,5H,6H,7H,7aH-pyrano [2,3-d][1,3]oxazol-2-yl]amino-6-(hydroxymethyl)oxane-3,4,5-triol) and Compound **2** (4-(hydroxymethyl)-6-[3,4,5-trihydroxy-6-(hydroxymethyl)oxan-2-yl]amino-hexahydro-[1,3]dioxolo [4,5-c]pyran-2-one) (Figure 6) were chosen as the best binders whilst being non-toxic, with binding energies of −8.9 kcal/mol and −8.4 kcal/mol, respectively. Pharmacokinetics and safety profiles of the virtual hits were also predicted using admetSAR 2.0 [52], ADMETlab 2.0 [53], and SwissADME [54], while scaffold optimization was performed using ADMETopt [55] to determine if better binding affinities can be obtained.

Given that molecular docking often makes use of rigid protein structure and only incorporates flexibility in the ligand structure, further computational analyses may be needed to increase the reliability of the results and obtain a better understanding of how a candidate inhibitor can affect the function of a target protein. Molecular dynamics simulations using Nanoscale Molecular Dynamics (NAMD) [56] were also conducted on *Ag*Tre bound to validamycin A, validoxylamine A, **67837201**, Compound **1**, and Compound **2** at 310 K to study protein-ligand interactions further while also including structural flexibility. Molecular simulation studies protein-ligand interaction and the overall complex energy landscape based on force fields to incorporate the effects of changing atomic positions, the presence of solvents, ions, and other physiological parameters. Incorporating both side chain and backbone flexibility allows a candidate ligand to explore better binding orientations and interaction with the receptor pocket, leading to better elucidation of binding affinities [49]. Based on RMSD, the binding of validamycin A, **67837201**, **10406567**, and Compound **1** led to minor fluctuations compared to validoxylamine A. As for Compound **2**, conformational changes of less than 3 Å were observed, indicating that the model orientation varies minimally. The average RMSD of the model bound to Compounds **1** and **2** showed lower values than when bound to validamycin A and validoxylamine A, indicating that the *Ag*Tre structure was more stable when interacting with the identified hits. From this, **67837201**, **10406567**, and Compound **2** were deemed as both excellent inhibitors of *Ag*Tre with desirable predicted safety profiles, showing future potential for malaria drug-based vector control. Interestingly, the computational results agree with in vivo studies of validamycin A and validoxylamine A against *Ag*Tre, wherein validoxylamine A resulted in the complete prevention of the development of normal adult pupae, while validamycin A resulted in only 11% of normal adult pupae being developed [57], indicating the validity of the experimental design. Hence, it would be interesting to establish the in vivo activities of the top hits, **67837201** and **10406567**, as well as the optimized hit, Compound **2**, in the future.

#### 3.1.3. Drug Repurposing of FDA-Approved Drugs for Antimalarial Drug Discovery

Despite the inclusion of computational tools and improvement of the overall drug discovery paradigm, high failure rates are still observed for leads, especially in the pre-clinical and clinical stages of the drug development process. Therefore, drug repurposing or repositioning has come out as an attractive strategy to minimize the risks currently associated with drug development. In this method, drugs that have already been approved for other indications are re-evaluated to determine their activities against other diseases. Furthermore, given that these approved drugs would already have pre-clinical and clinical data, including in vitro and in vivo activities, safety and toxicity properties, optimization data, and formulation, this can greatly expedite the drug discovery process [58,59].

In a recent study, 796 compounds from the DrugBank database [60,61] were virtually screened against 36 *P. falciparum* targets [25] using blind docking with QuickVina-W 1 [62]. After docking, prioritization of hits was conducted after rescoring with GRaph Interaction Matching (GRIM) [63] and determining ligand efficiency (LE) via surface efficiency index (SEI), binding efficiency index (BEI), and lipophilic efficiency (LipE) [64]. Ligands with molecular interaction similarity to the co-crystallized ligands and suitable predicted LE profiles were selected as hits. Ligand binding stability was analyzed for the best 25 protein-ligand complexes via molecular dynamics using GROMACS 2016 [65]. From the 20 ns trajectories obtained, the root mean square deviation (RMSD), the radius of gyration (Rg), and the number of H-bonds present were measured for each complex to monitor protein structure and ligand binding stability. While prazosin exhibited the best interaction energy profile, abacavir had the most favorable H-bond network and a good protein-ligand interaction energy.

**Figure 7:**
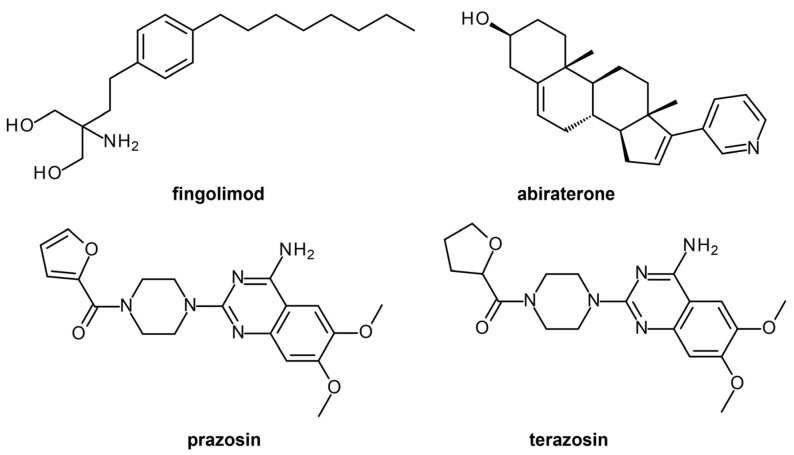
Top DrugBank compounds found active against *P. falciparum*.

Sixteen virtual hits were selected for the subsequent antiplasmodial assays, resulting in the identification of fingolimod (Plasmepsin 2 target), abiraterone (Calcium-dependent protein kinase 2 target), prazosin (Thioredoxin reductase 2 target), and terazosin (PfPK7 target) as actives (Figure 7).

### 3.2. Ligand-Based Methods

In the absence of validated 3D target structures, ligand-based drug design often helps in the identification of potential hits. Similar compound structures are assumed to have similar properties, and thus, using the structure of known small molecule antimalarial compounds, new ligands can be discovered and optimized to target malaria. Ligand-based methods include quantitative structure-activity relationship (QSAR), molecular fingerprints, 2D and 3D similarity screening, ligand-based pharmacophore modeling and screening, and ML models. Some of these have already been in antimalarial drug discovery in recent years (Table 1). Additionally, various artificial intelligence (AI) and ML methods have been employed in the discovery of novel drug molecules to process big data sets of compounds with known biological activities.

#### 3.2.1. Machine Learning and Virtual Screening for *P. falciparum* Protein Kinases

Calcium-Dependent Protein Kinases, or CDPKs, are regarded as the most valuable targets for regulating the life cycle of parasites. In particular, *Pf*CDPK1 and Protein Kinase G are both active during the sporozoite invasion of parenchymal liver cells [66,67]. In the study conducted by Lima et al. [68], shape-based and binary machine learning modeling was utilized to identify inhibitory compounds for *P. falciparum* CDPK1, CDPK4, and Protein Kinase 6 (PK6).

Advances in software and hardware have allowed for more complex calculations; therefore, machine (ML) and deep learning (DL) models are being developed for diverse fields. While an old technique already, machine learning has been reemerging as an essential strategy for drug discovery, design, and development. Compared to structure-based methods dependent on 3D structural information, machine learning uses pattern recognition, wherein physicochemical properties are explored to establish mathematical relationships between structure and activities. These relationships can then be employed to design novel compound structures and predict their properties. The major requirement of machine learning methods, however, is the availability of a large amount of data that can be used to “teach” a model that is being developed to increase its reliability and accuracy [69].

To apply machine learning models, data curation of the PubChem Bioassay databases was first completed. From this, the shape-based models were designed using multiple conformations of known ligands under the presumption that active and inactive compounds for the targets would be clearly differentiated using 1:36 (active:inactive) linear undersampling. The most potent against the kinases were loaded from the curated compounds in the ROCS v.3.2.2.2: OpenEye Scientific software [70] to be utilized as representative compounds to generate active and inactive compounds. These sets are evaluated and differentiated using statistical metrics of the Receiver Operating Characteristic (ROC) curve, the area under the curve (AUC), the Boltzmann-Enhanced Discrimination of ROC, and the Enrichment Factor. Aside from this, another ML model was generated by balancing *P. falciparum* 3D7 and W2 strains in a 1:1 (active:inactive) linear undersampling method, with the inclusion of multiple molecular fingerprints using RDKit (i.e., FeatMorgan, Molecular ACCess System structural keys (MACCS), AtomPair, Avalon) to differentiate the active from inactive compounds precisely. The application of molecular fingerprint seeks to simplify the molecular complexity often found in therapeutic molecules by incorporating topological, physicochemical, and substructure information within a few key features [71], making it indispensable in ligand-based computational techniques. Moreover, molecular fingerprinting has seen increasing applications in generating artificial intelligence (AI) and DL models in drug discovery. Random Forest algorithm was used to select the descriptors for model building [72]. Upon validation of the ML models with varying fingerprints, a consensus model was generated to obtain a model with a diverse range of chemical signatures, making predictions less susceptible to error.

Following this, virtual screening and experimental evaluation were conducted to validate compounds that can inhibit the reproduction of *P. falciparum* and exhibit multi-drug resistant strain inhibitory capabilities. Virtual screening of approximately 1.1 million compounds in the ChemBridge database (https://chembridge.com/) was performed to prioritize compounds that may exhibit antiplasmodial activity, which was then further assessed using Veber and Lipinski’s rules. The shape-based model was then utilized to filter molecules that could interact with the target kinases. Using the consensus ML model, the top 10% hits of each kinase were then filtered for an antiplasmodial inhibitory activity for the parasitic 3D7 and W2 strains. Molecular docking of predicted top 10 hits was also performed on the target kinases at the ATP binding site to study the binding interactions and identify the most potent ligand. Docking calculations predicted that LabMol-171 suitably interacts with the catalytic residues of all three target kinases, implying its potential to be a multi-kinase inhibitor. Furthermore, results from the in vitro cytotoxic assays using fibroblast monkey cell lines validated the anti-plasmodial effects of the top compounds. It was concluded that amongst the top 10 hits, compound candidates, LabMol-171, LabMol-172, and LabMol-181 were the most potent inhibitors of 3D7 and W2 strains, with LabMol-171 and LabMol-181 showing excellent in vitro inhibition, 70.02 ± 22.16% and 51.81 ± 23.16% ookinete conversion inhibition at 10 µM, respectively [68].

#### 3.2.2. Artificial Neural Network-Genetic Algorithm for Fusidic Acid Derivatives

Fusidic acid is a natural tetracyclic triterpene that can be obtained from fungi. Fusidic acid and its derivatives have established antimalarial activities [73], making it an attractive set of compounds to explore. A study by Azmi et al. [74] applied QSAR to generate a model that can be used to predict the antimalarial activity of novel compounds. QSAR is one of the conventional ligand-based methods that take advantage of the fact that the activity of a compound is related to its physicochemical properties. A statistical model is produced from this relationship, which can then be used to predict certain properties of a set of novel compounds, i.e., inhibition, activation, toxicity, etc. While it is a very useful technique in the absence of 3D structural target information, it does have several restrictions: (a) there should be sufficient bioactivity data with satisfactory activity range obtained from the same group or the same experimental protocol, (b) suitable selection of compounds for the training and test sets to avoid bias, (c) suitable selection of descriptors to avoid overfitting, and (d) proper model validation to evaluate the model’s applicability to the system and avoid bias to a certain scaffold [49].

**Figure 8:**
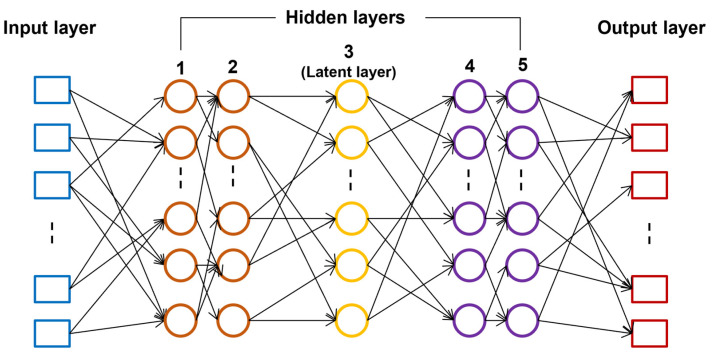
An example of artificial neural network architecture. A neural network consists of several layers including the input (blue squares), hidden (orange, yellow, and purple circles), and output (red squares) layers. Each square or circle can correspond to a neuron which contains information which it can pass to any of the other neurons in the next layer. The hidden layer receives data from the input layer and performs computations, leading to more data, which it will pass on to the outer layer neurons.

This study used 61 fusidic acid derivatives with established antimalarial activities for the QSAR model generation. MarvinSketch was first used to sketch the 2D structures of the compounds, and Open Babel [75] was employed to convert the files to 3D. The 3D structure of the compounds was used to acquire molecular descriptors, which contain information on the structure, topology, and electrostatic properties of the fusidic acid derivatives, using Mordred [76] and Padel [77]. From this, the training and test set was built with a 7:3 ratio. The IC50 was used as the experimental activity wherein inactive compounds (high IC50 values) were categorized as class 0, and active compounds (low IC50 values) were categorized as class 1. Genetic algorithm (GA) was used for the feature selection of the QSAR model, so descriptors with low standard deviation were removed. To further improve the model and remove bias, the correlation among all the descriptors was also analyzed to remove descriptors that contain similar information. After, an artificial neural network (ANN) was employed for the model development, creating 5 models containing different sets of descriptors. ANN has a similar architecture to the nervous system, particularly the brain, wherein (artificial) neurons are used to acquire the signal or information (input layer), process and pass the information to other neurons (hidden layer), and produce decisions (output layer) (Figure 8). ANNs can study nonlinear relationships found among parameters and help facilitate drug discovery processes [78,79]. As with traditional QSAR modeling, validation is performed to determine model accuracy and reliability and identify the best models. All five models underwent validation by calculating parameters including true positives (TP), false positives (FP), true negatives (TN), false negatives (FN), sensitivity (SE), specificity (SP), Precision (PR), accuracy (Q), and Matthew’s correlation coefficient (MCC). Further validation using y-scrambling analysis was conducted to confirm that no coincidental correlation was found. In the end, it was determined that model 3, consisting of 7 descriptors and internal and external accuracies of 0.96 and 0.92, respectively, was the best model out of the 5 that were initially generated [74].

ANN and other ML techniques have also been applied to other compound scaffolds used to target malaria [80,81]. However, with the current state of research for this tropical disease, it is evident that more studies utilizing AI and ML tools are needed to increase the information available for antimalarial compounds and potentially enhance the reliability of these methods in malaria drug discovery research.

## 4. Elucidation of Anti-Malarial Drugs Mechanism of Action

### 4.1. Artemisinin, Its Derivatives, and Combinatorial Therapy

**Figure 9:**
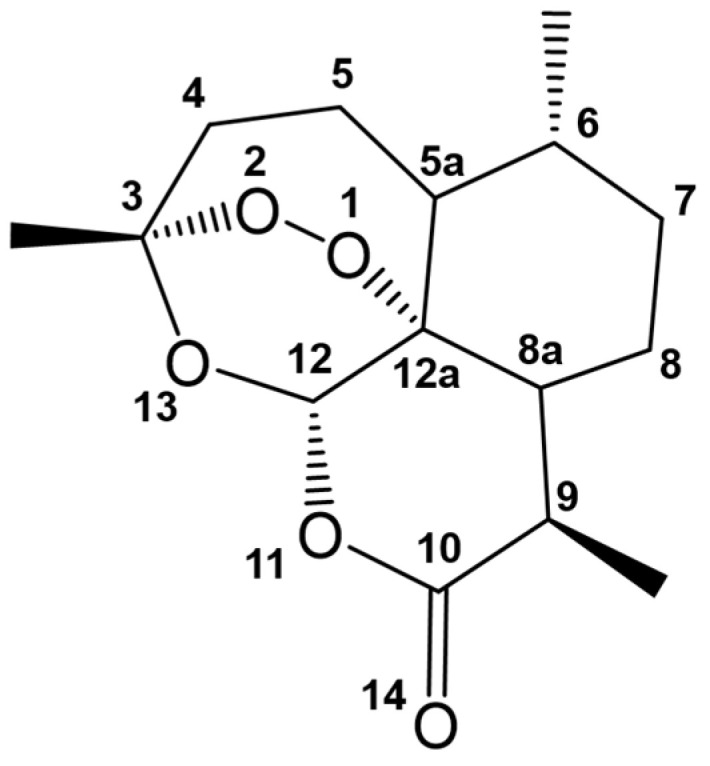
Structure of artemisinin, with atom numbering to emphasize O1 and O2.

An aspect of drug development that is equally important to identifying drug candidates is understanding their mechanisms of action. One of the well-known anti-malarial drugs is artemisinin. Multiple experimental pieces of evidence suggest different mechanisms of action of artemisinin. The common denominator among these mechanisms is the interaction of the endoperoxide of artemisinin (Figure 9) with the prosthetic heme group of hemoglobin. There usually is a one-electron transfer from the Fe^2+^ of the heme to the peroxide, producing free radicals or electrophilic intermediates capable of reacting with malaria-associated proteins [82,83,84,85,86,87]. A molecular docking study of artemisinin to heme showed that O1 at the endoperoxide linkage of artemisinin interacts with the iron center of the heme [88]. This docking study employed simulated annealing Monte Carlo simulation using AutoDock 2.4. As there were no available Amber force fields for iron, they used a combined Amber/MMFF force field to calculate the interaction energies. MMFF stands for Merck Molecular Force Field, which has van der Waals parameters for hydrates of Fe^2+^ [89] and must not be confused with the molecular mechanics force field. It highlighted how the structure of the heme used in docking studies could influence the calculated energies and distances between atoms. The choice of level of theory used for atomic charges is also important, concluding that HF/6-311G** and HF/3-21G are the better selections for the heme and artemisinin, respectively. A later DFT study contradicted this and calculated that it is O2 that interacts with the heme [87]. During the reductive decomposition of heme, the one-electron transfer will produce an anion on one of the oxygen atoms and a radical on the other. Their results presented lower energy for the transition state where O2 has a more negative charge, while O1 is a protonated radical having a neutral charge. The greater negative charge in O2 suggests that this oxygen interacts with the iron center of the heme instead of O1. This illustrates how DFT studies can be used to determine the more probable reaction route of a drug by looking at the transition state that requires less energy to be achieved.

**Figure 10:**
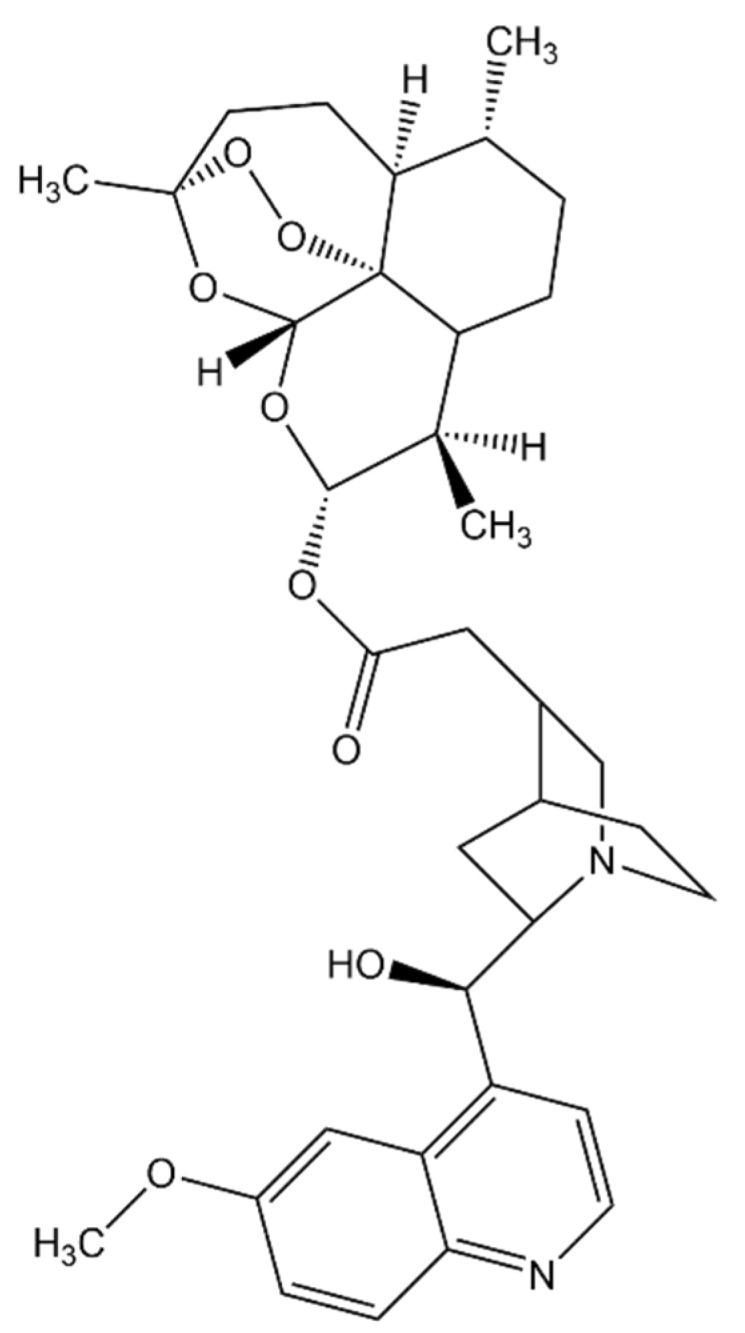
Hybrid molecule of artemisinin and quinine.

Another treatment strategy for malaria is combinatorial therapy, where two or more drugs are administered as they may have synergistic effects. Hybrid drugs can also be developed where the components are covalently linked to each other instead of co-formulating two or more drugs into a single tablet [90]. Artemisinin and quinoline-containing molecules are antimalarial drugs that can inhibit heme detoxification [91]. One of the quinoline drugs, quinine, is known to inhibit nucleic acid synthesis [92] in *P. falciparum*. However, the exact mechanism of the drug still needs to be fully elucidated. An experimentally-synthesized hybrid molecule of artemisinin and quinine (Figure 10), including its different analogues, was studied in silico to evaluate its binding mode and affinity to Fe(II)PPIX [90]. Unlike the previous docking study of lone artemisinin with heme, this hybrid drug-heme docking study [90] fixed the charge of iron to +2 and used the Optimized Potentials for Liquid Simulations (OPLS)-2005 force field. The model of the heme assumed a planar geometry with the carboxylic acid portion, providing a potential steric hindrance to the drug. Despite this, artemisinin-quinine could bind to the heme via one of the oxygens of the endoperoxide linkage. The O2 of the linkage has the shortest distance to Fe, supporting the DFT study discussed earlier. In general, the binding energies calculated for the hybrid drug and its analogues were greater in magnitude than those of artemisinin alone. It may be a poor comparison as different force fields, docking methodologies, and binding energy calculations were employed. It is, therefore, noteworthy to know whether binding energies or conformations can influence the synergy between artemisinin and quinine.

### 4.2. Falcipains as Drug Targets

Falcipains are a family of cysteine proteases that antimalarial drugs can also target. There are three known subclasses: FP-1, FP-2, and FP-3, which have different roles in the infection of *P. falciparum*. Gene knockout studies have shown that only FP-2 and FP-3 play an essential part in the survival of the parasite [93], making them potential targets for drug candidates. FP-2 and FP-3 are hemoglobinases found in the vacuole of *P. falciparum*, which is essential for the acquisition of amino acids by the parasite from the cleavage of proteins in erythrocytes. Experiments have shown that FP-2 preferentially cleaves hemoglobin during its early trophozoite stage, while the late trophozoite stage shows a marked increase in the cleavage of membrane skeletal proteins [94]. Either way, FP-2 and FP-3 cause hemolysis and, thus, play a significant role in the pathogenesis of malaria. As such, inhibiting its activity is a potential treatment mechanism.

**Figure 11:**
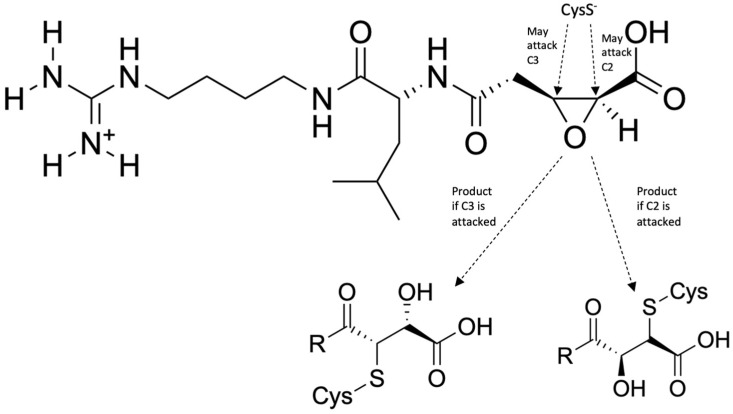
General mechanism of FP–2 inhibition by E64 [30]. Redrawn and reproduced with permission from Biochemistry.

An inhibitor of falcipain, studied using molecular dynamics, is the compound N-[N-(1-hydroxycarboxyethylcarbonyl)leucylaminobutyl]guanidine, also known as Epoxysuccinate E64 [30]. This compound has two possible mechanisms of inhibition, both of which involve the attack of Cys42 on FP-2 to either C2 or C3 of the epoxide, leading to ring opening and protein complex stabilization (Figure 11). Its mechanism of action for inhibiting FP-2 was elucidated via MD simulation using hybrid AM1d/MM and M06-2X/MM potentials. The side chains of FP-2 were optimized at pH 5.5 using the empirical PROPKA 3.1 program, simulating the pH inside the food vacuole. The protein, except for the quantum mechanics (QM) region, along with the water molecules, was described by the OPLS-AA and TIP3P force fields, while the semi-empirical Hamiltonian AM1d described the QM region. The fDYNAMO library was used for all quantum mechanics/molecular mechanics (QM/MM) calculations in this study. The results of this study indicate that the attack of Cys42 on the alpha-chain of FP-2 to C2 of the epoxide is kinetically favored by approximately 1.3 kcal/mol (5.44 kJ/mol). Still, the attack of Cys42 to C3 is thermodynamically favored by 47.7 kcal/mol (200 kJ/mol). This means that the attack on C3 yields a more stable enzyme-inhibitor complex and, thus, yields better binding and more potent inhibition.

**Figure 12:**
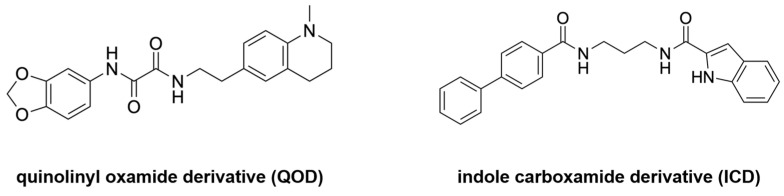
Structures of QOD and ICD. Redrawn and reproduced with permission from Frontiers in Molecular Biosciences [31].

Another example of falcipain inhibition is exhibited by N-(2H-1,3-benzodioxol-5-yl)-N’-[2(1-methyl-1,2,3,4-tetrahydroquinolin-6-yl) ethyl]ethanediamide, a quinolinyl oxamide derivative (QOD), and N-{3-[(biphenyl-4yl carbonyl) amino]propyl}-1H-indole-2-carboxamide, an indole carboxamide derivative (ICD) (Figure 12) [31]. These molecules have been observed to have a strong inhibitory property against FP-2 and FP-3. The mechanism of inhibition using these molecules was elucidated via MD simulations using GROMACS 2021.3. The structure of FP-2 and FP-3 were obtained from RCSB PDB, while the structures for QOD and ICD were obtained from the MolPort database in SDF format and then converted to PDB format. The structures were then used to generate an Amber forcefield using AmberTools22. The compounds were then solvated using the TIP3P water model, neutralized with 150 mM NaCl, and the ligand parameters defined with the help of the GAFF forcefield. The results of this study show that both QOD and ICD ligands occupy the same binding pocket at the active site of FP-2. In addition, they are coordinated by nearly the same residues. Specifically, QOD forms interactions with residues Q36, N38, A157, W206, Q209, and W210. This interaction is also highly stabilized by a hydrogen bond between residues Q209 and W206 with an oxygen atom on the dioxanyl ring and the carbonyl oxygen of the ethanediamide ligand backbone. In the same vein, ICD interacts with the residues Q36, A157, W206, and W210. In addition, hydrogen bonding is observed between the formamide carbonyl oxygen of the ligand and residues D35 and K37, with an additional hydrogen bond formed between a side chain of oxygen on D35 and the nitrogen atom on the indoyl ring of ICD. In contrast, QOD and ICD are not fully embedded in the active site of FP-3, but the interactions between them are enough to block substrate binding. The ligand QOD interacts with FP-3 via a hydrogen bond between a nitrogen atom on the side chain of N86 and the amide oxygen of the ethanediamide backbone. Two additional hydrogen bonds form between backbone oxygen on Y90 and N and O atoms in the ethanediamide. QOD also forms interactions with C51, H183, Y93, I94, N96, S158, A184, and E243. ICD, on the other hand, forms a hydrogen bond only between a side chain oxygen of D163 and the nitrogen atom of the indonyl substituent of the ligand. It also forms interactions with A46, A166, N182, H183, W215, and W219. In essence, both QOD and ICD inhibit hemolysis by preventing its access to the active site of FP-2 and FP-3.

### 4.3. Interaction of Antimalarial Drugs with Serum Albumin

The transport of antimalarial drugs via blood circulation is another aspect that can be investigated in the drug discovery process. Serum albumin is a blood plasma protein that has an essential role in the in vivo circulation and distribution of endogenous ligands and exogenous drugs [95,96]. Strong interaction between the carrier protein and the drug will lower the availability of the drug in the plasma, while a weak interaction will distribute the drug poorly in vivo. Thus, the interaction of antimalarial drugs with plasma proteins is important in their pharmacodynamics and pharmacokinetics. In this section, different drugs that are evaluated for their binding interactions with serum albumin will be discussed.

**Figure 13:**
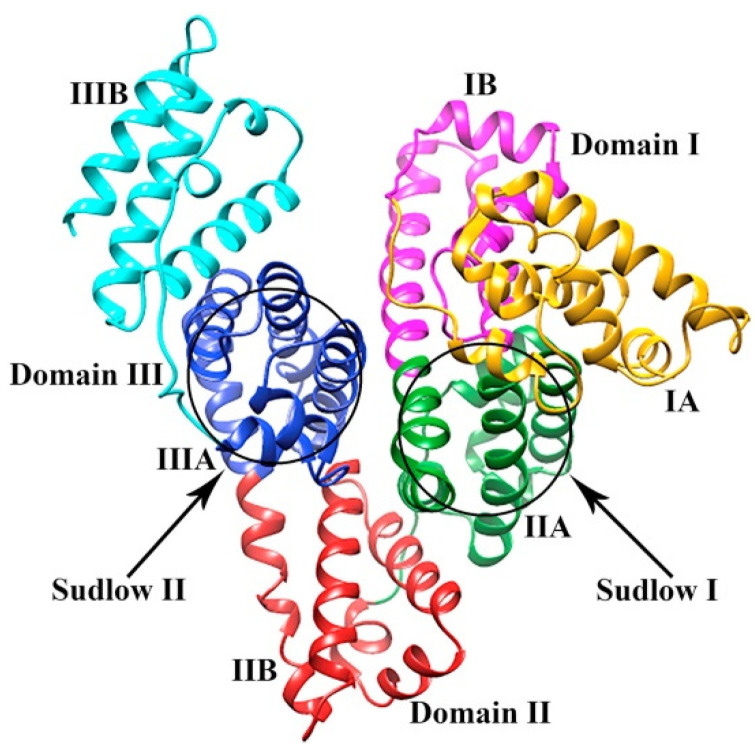
Structure of human serum albumin showing the different domains and the locations of Sudlow binding sites I and II. Reproduced with permission from the Journal of Luminescence [100].

Human serum albumin (HSA) is a carrier protein with 585 amino acids that can transport hydrophobic molecules in plasma [97]. HSA has three homologous α-helical domains (Figure 13), further divided into subdomains A and B. In these domains are two primary ligand binding sites located in subdomains IIA and IIIA, also known as Sudlow’s Site I and Sudlow’s Site II, respectively [98,99].

**Figure 14:**
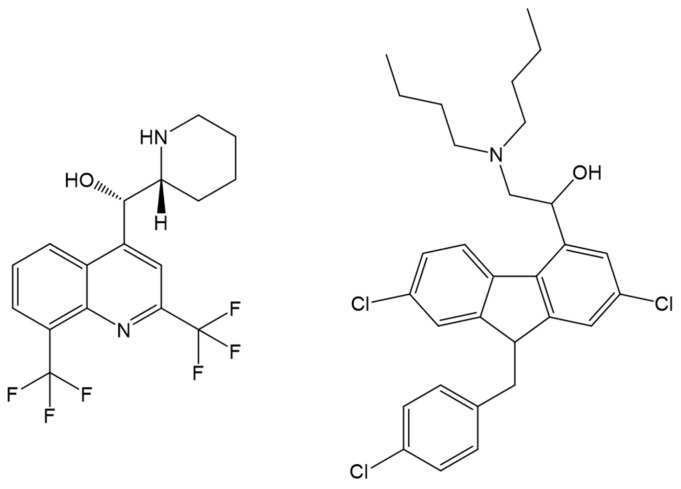
Structures of mefloquine and lumefantrine, which were investigated for their binding with human serum albumin for blood transport.

Mefloquine (Figure 14) is an FDA-approved anti-malarial quinine derivative drug that attacks *Plasmodium* during the blood-stage of the parasite’s life cycle [30,31,32]. In the study of Musa and coworkers [101], the docking calculations supported that Mefloquine prefers to bind to Site I based on a more negative lowest binding energy upon binding to Site I (approximately −27 kJ mol^−1^) than to Site II (approximately −23 kJ mol^−1^). Amino acids Tyr150 and Arg257 were seen to form hydrogen bonds with mefloquine [101]. Another drug, Lumefantrine (Figure 14), is used in combination with other antimalarial drugs to treat malaria. In a similar study by the same group, Lumefantrine was found to have a preferred binding site to HSA in Site I [102]. Furthermore, there were more hydrogen bonding interactions (one with Cys448) and a greater hydrophobic effect when the drug binds to Site I [102]. These were also observed in the binding characterization of Mefloquine with HSA [101]. These agreed with the observed preferential binding of Lumefantrine to Site I in site marker fluorescence displacement experiments. The average free binding energy of binding Lumefantrine to Site I calculated using molecular docking studies (−29.83 kJ mol^−1^) is close to the obtained free binding energy using fluorescence titration (−27.31 kJ mol^−1^). However, it is essential to note that the experimental value is even more comparable to the calculated binding energy to Site II (−26.95 kJ mol^−1^) [102]. Determining the binding site preference based on average binding energy calculations is more reliable than merely comparing the lowest binding energy among the sampled conformations. MD simulations might be helpful in this aspect to increase the sampling size of the complexes and determine the stability of the drug in both sites.

**Figure 15:**
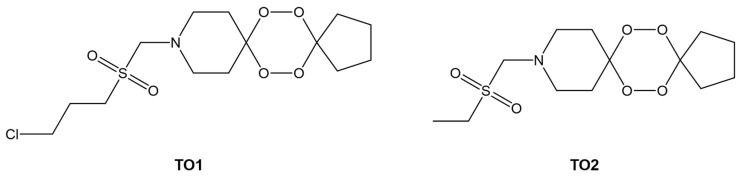
Artemisinin analogues, TO1 and TO2, with potential antimalarial activity. Redrawn and reproduced with permission from Yadav, P., Sharma, B., Singh, P., and Awasthi, S.K., Interaction between the antimalarial drug dispirotetraoxanes and human serum albumin: A combined study with spectroscopic methods and computational studies, published by ACS Omega, 2020 [103].

The transport and pharmacokinetics of the developed artemisinin analogues, TO1 and TO2 (Figure 15), will be better understood by looking at their binding with HSA. Molecular docking studies revealed that TO1 and TO2 bind better to Sudlow’s Site I, similar to Lumefantrine and Mefloquine. Furthermore, the conformers with the lowest binding energy for each analogue showed that hydrogen bonding (with Asp324 and Lys212) and hydrophobic interactions are also significant in the binding.

Analogues of artemisinin, referred to as TO1 and TO2 in this paper (Figure 12), were synthesized and developed by the group of Awasthi [103] as lead candidates of antimalarial drugs that carry the vital endoperoxide. DFT calculations were conducted to determine which is more reactive. Molecules with smaller HOMO–LUMO gaps were correlated to more reactive molecules [104]. The smaller gap for TO1 suggests that this is more reactive than TO2 and supports the more excellent antiplasmodial activity of TO1 in vitro [105].

**Figure 16:**
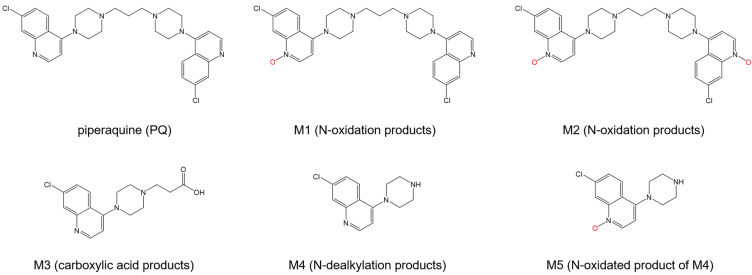
Chemical structure of piperaquine and its derivatives after biotransformation. Oxygen atoms involved in the quinoline N-oxide functional group are highlighted in red.

Piperaquine (PQ) is an antimalarial drug used therapeutically in combination with artemisinin [106]. Its metabolites, as it is biotransformed, were seen to have antimalarial activity (Figure 16) [107]. Among PQ and its five metabolites, only M3 has comparable free binding energy values for both sites (−32 kJ mol^−1^ for Site I, −33 kJ mol^−1^ for Site II). The other metabolites and PQ all have favorable binding energies for Site I, ranging from −28 to −41 kJ mol^−1^. However, only M3 and M5 reported binding energies for both sites, as the site marker experiments needed to provide their preferential binding sites clearly. In addition to the hydrogen bonding and hydrophobic effects observed from the previously discussed antimalarial drugs, the docking conformations exhibited π-π stacking interactions and cation-π interactions due to the presence of aromatic rings in PQ and its metabolites [108].

**Figure 17:**
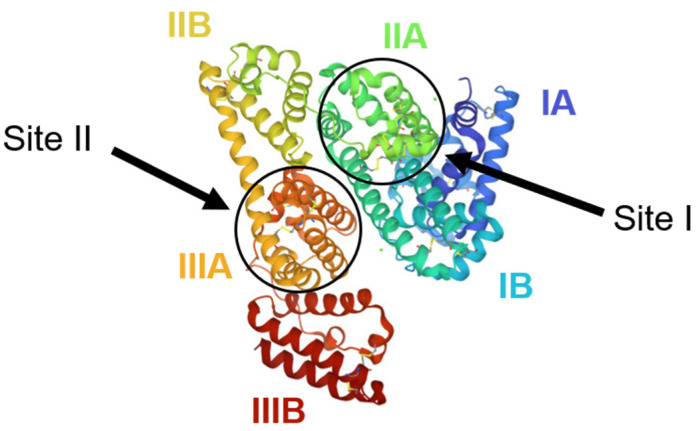
Crystal structure of bovine serum albumin and its binding sites, labeled Site I and Site II.

Bovine serum albumin (BSA) is another carrier protein derived from cows. The mature protein contains 583 amino acids, with 75.6% sequence identity with HSA [109]. Like HAS, BSA has three homologous domains (I–III), with two subdomains A and B (Figure 17). It is more commonly used as a model protein for evaluating binding between drugs and serum albumin because it is less expensive than HSA.

**Figure 18:**
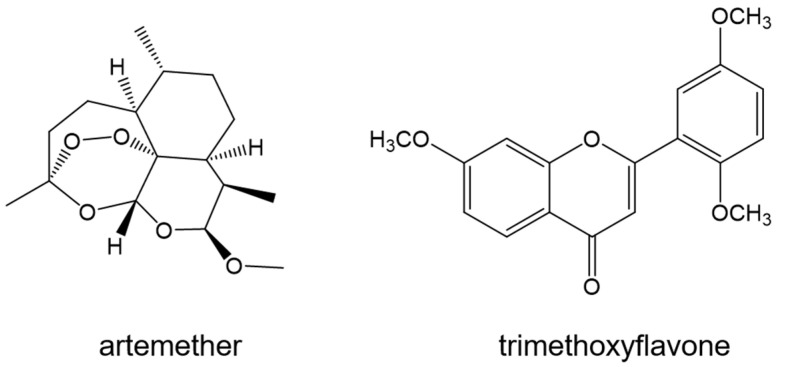
Structures of artemether and trimethoxyflavone.

Artemether (Figure 18) is an artemisinin derivative of the peroxide sesquiterpenoides drug class. It is used as a treatment against multiple resistant strains of *P. falciparum*. Molecular docking was conducted, in tandem with site marker competitive experiments, to identify the binding sites of artemether. Using Autodock 4.2, the drug was docked to the BSA based on the binding sites of phenylbutazone and ibuprofen, which are drugs known to bind to Sites I and II, respectively. The binding of artemether to the hydrophobic cavity in Site II (−32.40 kJ mol^−1^) has more negative free energy as compared to its binding to Site I (−28.01 kJ mol^−1^) [110]. This is different from the binding mechanism of the four drugs discussed earlier. While all these drugs were surrounded with hydrophobic residues, no hydrogen bonding interactions were seen in artemether, unlike in the Mefloquine and Lumefantrine.

GOLD, which stands for Genetic Optimization for Ligand Docking [111,112,113], is a docking program based on a genetic algorithm that can also be used in docking ligands to proteins. AutoDock, conversely, uses a Lamarckian genetic algorithm combined with an empirical force field [114]. GOLD v3.2 was used to evaluate the binding mode of an antimalarial drug, tri-methoxy flavone (TMF) (Figure 18), to HSA. This drug binds to Site II of the HSA (−26 kJ mol^−1^) with three hydrogen bond interactions, stabilizing the complex, involving Asn391, Arg410, and Tyr411 [115]. However, the study should have mentioned if TMF was attempted to be docked to Site I.

As summarized in Table 2, antimalarial drugs may also be screened using molecular docking by evaluating their binding interactions with sites of either BSA or HSA. The binding free energy ranges from −26 kJ mol^−1^ to −32.40 kJ mol^−1^, which may be a good benchmark for transporting antimalarial drugs via serum albumin. It should be noted that when conducting molecular docking of antimalarial drugs to serum albumin, it is better to evaluate the binding free energy for both sites and identify the present hydrogen bonding interactions, as these may be important in the binding characterization of the drug. Based on the studies reviewed, it has been highlighted that the hydrophobic nature of the cavity in the Sudlow sites is significant in binding antimalarial drugs, suggesting the critical role of hydrophobic regions in these small molecules.

### 4.4. Molecular Mechanism of Anti-Malarial Drug Resistance

One of the pressing problems in therapeutic research is the development of resistance against drugs that are commercially available and easily accessible. Most antimalarial agents were reported to be ineffective against drug-resistant strains of *P. falciparum* [116]. This led scientists to quickly develop new antimalarial drugs and look for novel drug targets [117]. Concomitantly, molecular mechanisms of this resistance should be elucidated to understand better how we can develop drugs that will minimize resistance development.

Chloroquine (CQ) (Figure 1) is a well-known antimalarial drug that prevents heme detoxification by binding to Fe(II)-protoporphyrin-IX (Fe(II)PPIX), thereby killing the parasite with its own waste [118]. Surprisingly, no computational studies were conducted on the interaction of chloroquine with heme and hematin. The mechanism of action of this drug was mainly unraveled from experimental results [119]. As of writing, the only computational study we have seen looks at the resistance mechanism of *P. falciparum* against chloroquine. As there is no available solved structure for *P. falciparum* chloroquine resistance transporter protein (PfCRT), homology modeling was conducted using MODELLER, and the selected models were validated using PROCHECK and Swiss-Model servers. A Lys to Thr mutation (K76T) in PfCRT was thought to increase the resistance of the parasite against chloroquine; thus, the mutant protein was also modeled. Upon molecular docking, the protonated forms of the chloroquine did not bind to the wild-type protein, supporting the hypothesis that electrostatic repulsion between positively charged lysine and protonated chloroquine will result in unfavorable binding. No alternative conformations resulted in a favorable interaction between the two molecules. On the other hand, the protonated chloroquine became bound to the mutant protein due to the loss of repulsion. The binding energy between the neutral form of chloroquine and mutant PfCRT was even greater than that of the wild-type [118]. The digestive vacuole of the parasite is where heme detoxification occurs, making it a good target for chloroquine. Whether PfCRT is a channel or a transporter, the enhancement in the binding affinity of both forms of chloroquine to the mutant PfCRT makes it easier for the parasite to remove the unwanted drug from its system, enabling drug resistance.

Pyrimethamine is an antimalarial agent that inhibits dihydrofolate reductase (DHFR), a crucial enzyme for replicating the malaria parasite within the human body [120]. The protein is critical in the folate biosynthesis pathway, essential for the parasite’s survival. Upon entering the host, the malaria parasite takes up folate from the human body. It relies on DHFR to convert dihydrofolate (DHF) to tetrahydrofolate (THF), which is necessary for its growth and replication. Antifolate drugs such as pyrimethamine and trimethoprim target DHFR and are commonly used to treat and prevent malaria. However, the effectiveness of these drugs has been compromised in recent years due to the emergence of drug-resistant strains of the malaria parasite, which possesses DHFR mutations, reducing its binding affinity to antifolate drugs [121].

MD simulations may be combined with other computational techniques to understand drug resistance mechanisms. For example, dynamic residue network analysis (DRNA) was combined with MD simulations to provide some mechanistic insights into the pyrimethamine drug resistance against *P. falciparum* [122]. Four-point mutations were selected (N51I, C59R, S108N, and I164L), and different protein mutants, ranging from single mutants to quadruple mutants, were generated using homology modeling. Molecular docking results showed that the S108N mutation induced steric clash to pyrimethamine. In contrast, N51I and I164L mutations increased the size of the active site, which decreased the binding affinity of small inhibitors [122]. Free energy calculations were conducted using Molecular Mechanics Poisson–Boltzmann Surface Area (MM/PBSA) method [123]. As the number of mutations increases, the total binding free energy becomes more positive relative to the wild-type (−127.2 kJ mol^−1^). DRNA is a tool that identifies significant residues involved in intra-protein communication by computing two parameters, average shortest path and betweenness centrality (BC), as a running average across an MD trajectory [124,125]. BC indicates the frequency of residue participation in the shortest paths between all residue pairs. Different residues have enhanced or decreased BC values for the wild-type and quadruple mutants, serving as molecular fingerprints that may give clues on the degree of resistance. A specific residue, Cys59, was identified to have a high average BC value for the wild-type strain, which may be affected in the strains that contain mutations at Cys59 [122].

Steric constraints in the DHFR, caused by an S1089 mutation, resulted in resistance to antifolate drugs [126]. A lead antimalarial compound, P218, binds similarly to both quadruple and double DHFR models and wild-type counterparts (−34.90, −34.15, and −34.93 kcal mol^−1^, respectively) but differently to human DHFR (−22.14 kcal mol^−1^), suggesting that developing a more selective DHFR inhibitor that only targets the parasite DHFR is possible. Evidence from molecular dynamics also shows that the differentiating hydrogen bond between pfDHFR and human DHFR (hDHFR) is the terminal carboxyl group of P218 hydrogen bond interactions with Arg122. Per-residue decomposition study in H-bonds shows that P218–hDHFR interactions (which contain Arg122 homolog) do not express favorable interactions compared to mutated and wild-type counterparts [127].

**Figure 19:**
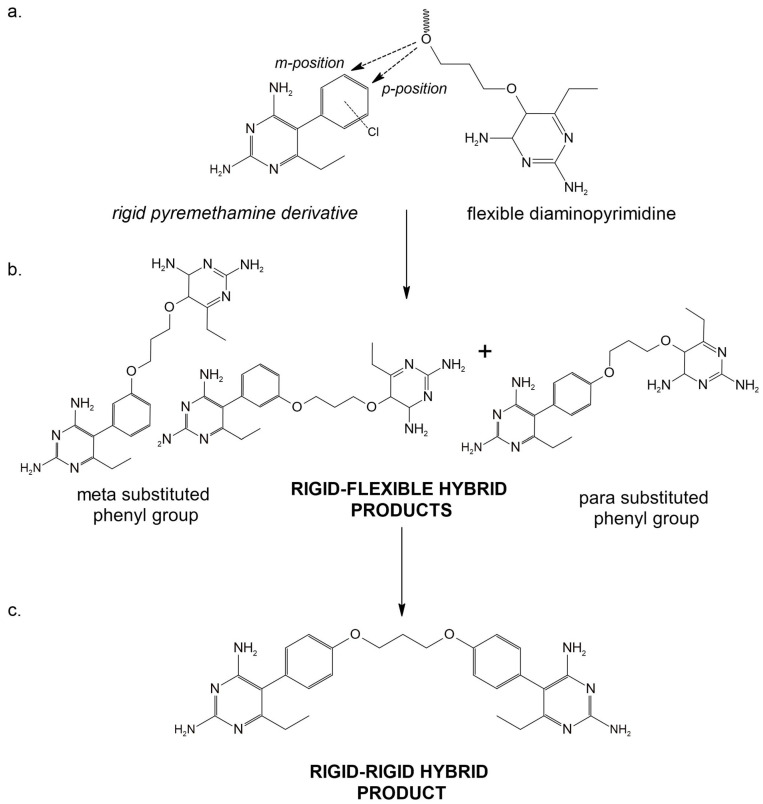
The general reaction of hybrid product formation. (**a**) The approach is to create rigid-flexible products, shown in (**b**), from pyrimethamine derivatives and a flexible diaminopyrimidine compound directed into the meta- and para-phenyl substituent position. (**c**) Rigid-rigid hybrid compounds can also be developed by condensation reaction. Redrawn and reproduced with permission from ACS Medicinal Chemistry Letters [128].

The threat of antifolate resistance arising from key mutations, such as S108N, suggests that a new approach is needed to address the attenuation of the pharmacokinetic activity of antifolate drugs. Inhibitors that contain rigid and flexible pharmacophores were developed and predicted to be effective and highly selective to DHFR [128]. Introducing flexible moieties at either meta- and para-phenyl substituent end (Figure 19), as well as developing a hybrid rigid bearing end, displays remarkable selectivity and inhibition activity to both wild-type and pyrimethamine resistant DHFR strains based on in vitro and in vivo assays.

### 4.5. Other Potential Targets for Inhibition of Resistant Malarial Strains

The application of hybrid compounds was further proven effective in antimalarial scenarios. Other than DHFR inhibition properties, compounds such as quinoline hybrids were also synthesized and assessed in both in vitro and in silico experiments [29]. One potential drug target is the glutathione reductase enzyme (GRE). The malaria parasite is sensitive to oxidative stress caused by reactive oxygen species, which is why antioxidants are vital for the malarial parasite in its host. GRE plays a crucial role in the redox metabolism of *P. falciparum* by reducing oxidized glutathione (GSSG) to its reduced form (GSH). This conversion helps maintain the redox homeostasis of the parasite and protects it from oxidative stress. Inhibition of glutathione reductase activity can lead to an accumulation of GSSG and ultimately cause oxidative stress, which can be lethal to the parasite making GRE a viable therapeutic target [129,130].

Dihydropyrimidinone–quinoline hybrids were synthesized and subjected to in vitro antimalarial sensitivity and inhibition activity assays utilizing K1 chloroquine (CQ)-resistant and NF54 CQ-sensitive strains [29]. Quinoline hybrids with amino functionalities show significant inhibition against *P. falciparum* strains. These are also comparably potent with CQ in antimalarial assays involving CQ-sensitive strains. Remarkable inhibition activity was also seen for K1 CQ-resistant strains with IC_50_ ranging between 421 and 567 nM concentrations. In silico techniques were employed to assess the binding activity and molecular dynamics of the effective quinoline hybrids. These were further optimized for competitive inhibition of GRE against the flavine adenine dinucleotide (FAD) cofactor. Researchers came to this rational optimization approach to ultimately design more potent hybrid products based on compounds with significant activity assessed from inhibitory assays conducted in vitro. This also provides substantial information for future researchers on designing potent antimalarial drugs. Moreover, this research has implicitly demonstrated the versatility and significance of in silico methods, particularly in their ability to facilitate post-assay optimization. This could enable time and cost efficiency for subsequent optimization experiments and improved lead generation.

## 5. MD Simulations of Peptide Immunogens for Vaccine Development

The onset of malaria brought about by *P. falciparum* may be prevented through peptide sequences that elicit the body’s immune response [131]. These peptide sequences must therefore be antigenic, capable of binding to B-cell receptors on antibodies and activating adaptive immunity, which should offer lasting protection against the disease. These antigenic peptide sequences are known as B-cell epitopes (BCEs), which bind to structures on the antibodies known as paratopes [132]. These epitopes may be identified from different accessible proteins in *P. falciparum*. Given the multitude of proteins that can be targeted and the number of amino acid residues on each protein, computational methods are used to aid this process. This happens through B-cell epitope prediction and mechanistic elucidation of the epitope-paratope binding [133].

### 5.1. Circumsporozoite Protein

The protein from which the most promising epitopes come from in *P. falciparum* is the circumsporozoite protein (PfCSP) found on the pathogen surface in the sporozoite stage of the parasite [134]. This protein is crucial for parasite motility, sporozoite development, and the ability to invade liver cells. Eliciting an immune response at this stage is the primary focus since it can prevent infection and eventual transition to symptomatic malaria. There are two significant peptides obtained from epitopes of this protein that elicit a strong immune response: KQPADGNPDPNANPNV (junctional peptide) and NPNANPNANPNA (NANP peptide) [135]. When these peptides were injected into transgenic mice, they produced a total of 2588 antibodies. Out of these, only 15 antibodies yielded a positive result in ELISA, and only two of these (mAb667 and mAb668) displayed significant anti-malaria activity.

The binding of the junctional peptide with the fragment antigen-binding region (Fab) of mAb668 was then elucidated via molecular dynamics (MD) simulations [135]. This was performed to determine precisely which residues contribute to the epitope-paratope binding the most. The NANP peptide was no longer analyzed since the junctional peptide also contains a NANP sequence. At the same time, Fab667 was also excluded since it did not show significant binding with the junctional peptide. These MD simulations were performed using the Protein Preparation Wizard in Maestro (Schrödinger) at neutral pH. A cubic box of TIP4P-EW water and 150 mM KCl with a 10 Å buffer in AMBERTools was used to solvate the models of the MD simulation. Heating, equilibration steps, and energy minimization of the system were conducted via AMBER16. The molecular mechanics/generalized Born solvent accessibility (MM/GBSA) approach was used to estimate the energy of peptide bonding to Fab668. To dissect the specific binding behaviors of different segments of the junctional peptide, MD simulations were conducted using five epitope registers containing either one or two repeats suspected to bind Fab668. These are: ^1^NPDPNANP^8^, ^1^NANPNVDP^8^, ^1^NVDPNANP^8^, ^1^NANPNANP^8^, and ^1^PADGNPDP^8^. Among these, ^1^NPDPNANP^8^ has the lowest root mean square fluctuation (RMSF) from the MD simulations, which corresponds well with the observed crystal structure of the complex of Fab-668-junctional peptide. It may also be caused by the conformational restriction given to Asn^1^ by Pro^2^, something non-existent in the other epitope registers. A noteworthy observation is that the ^1^NPDP^4^ has a much higher RMSF than ^5^NANP^8^ when present in the same peptide. This indicates that the binding of the second repeat has a more significant impact on the overall binding than the first. The substitution of ^5^NANP^8^ for ^5^NVDP^8^ also increases the RMSF of individual amino acid residues by 1 Å, suggesting preferential binding with Fab-668 with the first repeat. Comparisons of relative ΔG contributions via (MM/GBSA) also provide insight into residue importance. In all five epitope registers, all amino acid sequences show differences except for Asn^5^ and Pro^8^. Considering this, the results show that neither Asn^1^ nor Pro^1^ contributes to the overall binding. In contrast, positions two and three contribute more than position one but less than positions four to eight, with position three slightly preferred for Asn than Asp. Pro^4^ was also observed to contribute more than Gly^4^, indicating unfavorable Fab-668-^1^PADGNPDP^8^ binding. There is also a slight preference for Val^6^ and Pro^6^ compared with Ala^6^, but the most drastic change is observed when Asn^7^ is replaced by Asp^7^, which causes an absence in contribution to favorable binding ΔG. This may be due to the lack of hydrogen bonding with Fab668 experienced by the negatively charged Asp^7^, which leads it to take an out-of-pocket orientation, in contrast to the in-pocket orientation taken by the neutral Asn^7^. The absence of this H-bond network with Fab668 is the most probable reason that the NVDP repeats on the junctional peptide are unlikely to bind to the NANP paratopes on Fab667 and Fab668, and thus eliciting a weaker immune response compared with the NANP peptide.

### 5.2. Ring-Infected Erythrocyte Surface Antigen

Another protein of interest where significant epitopes can be found is the ring-infected erythrocyte surface antigen (RESA). This protein likely facilitates the malaria parasite’s survival in living organisms due to its omnipresence in field isolates of *P. falciparum* [136]. This protein is stored in dense granules, which are apical organelles in individual merozoites, after being produced in the final stages of the development of the schizont [137]. RESA is secreted into a parasitophorous vacuole (PV) after the host cell rupture and subsequently reinvades a new erythrocyte, where it interacts with erythrocyte spectrin to stabilize its cell membrane, possibly aiding in its repair after invasion [138].

Virtual screening via immunoinformatics has been applied to determine the most immunogenic B-Cell epitopes found on the RESA protein. There are 10 B-Cell epitopes of interest: TQANKQELANI, YGYDGIKQV, RWYNKYGYDGIKQV, SSSSGVQFTDRCS, KDFTGTPQIVTLLR, NLYGETLPVNPY, AIKKTKNQEN, TEEEKDDIKNGKDI, SCYNNNFCNTNG, and NNKNDDSYRYD [139]. These B-Cell epitopes were virtually screened for their antigenicity via the computational tool VaxiJen v2.0. This software possesses a 78.0% prediction accuracy and is based on the physicochemical features of a protein [140]. VaxiJen v2.0 screens epitopes by assigning them antigenicity scores, with a higher score indicating better antigenicity. Using this tool, the NLYGETLPVNPY epitope obtained the highest antigenicity score (1.8128), with the TEEEKDDIKNGKDI epitope being a close second (1.2106). However, other relevant parameters, such as allergenicity and water solubility, must also be considered when screening for these epitopes to obtain the whole picture. Allergenicity was computationally determined via AllerTOP v2.0, a tool based on comparing the sequences of allergens and non-allergens while incorporating the k-nearest neighbors (kNN) concept [141]. On the other hand, the solubility in water of the screened epitopes was evaluated via Protein-Sol. This server predicts the solubility of proteins obtained from *Escherichia coli* in a cell-free expression system based on the protein sequence [142]. Using these tools, the NLYGETLPVNPY epitope was found to be allergenic and have poor water solubility. In contrast, the TEEEKDDIKNGKDI epitope was found to be non-allergenic and highly soluble in water. Thus, despite its slightly lower antigenicity score, the TEEEKDDIKNGKDI epitope was the optimal B-Cell epitope found on RESA via this virtual screening using immunoinformatics.

## 6. Conclusions

Antimalarial computational drug discovery continues to be an important field of research, given that malaria still affects millions of people globally. One of the major hurdles in this field is still the complexity of the parasite and its lifecycle, paired with the emergence of resistant strains that have started to evade current treatment strategies, and thus, requiring an urgent search for new therapeutic agents. Another challenge is the limited amount of data and resources for this field since this disease is often endemic to developing countries.

Even with these difficulties, the ongoing antimalarial research, combined with the constant advances in technology and computational tools, provides an excellent outlook for developing new antimalarial agents. With the availability of high-performance computing facilities, computational and ML methods, and new AI tools, more and larger data sets can now be produced and/or analyzed for malaria. Current pharmaceutical research, both in academia and the industry, also often integrates computational and wet lab tools. For example, ML algorithms can help produce models that can predict drug properties and activities, as well as optimize potential leads. AI tools can then be used to analyze immense amounts of data, facilitating the identification or design of new antimalarial hits. These data analytics tools are best utilized when the drug discovery process is still in the early stage and the number of potential drug targets needs to be reduced greatly. Once the number of compounds is manageable, which is highly dependent on the available computational resources, molecular docking techniques can be employed to predict the binding affinity of the compounds to the protein target. The binding stability of the docked conformations can then be assessed using MD simulations. Furthermore, drug transport in the bloodstream can be predicted by docking compounds to serum albumin, followed by MD simulations. In general, computational tools help expedite the drug discovery process by identifying which compounds are to be prioritized for the expensive experimental assays, allowing for more rational use of resources.

Overall, the future of antimalarial drug discovery research is positive. With continued resource investment and advances in research technologies, there is hope that several novel and highly effective antimalarial drugs can be discovered, designed, and developed in the following years to help alleviate the global burden of malaria.

## Figures and Tables

**Table 1:** Recently published computational antimalarial drug discovery studies.

Drug Target	Computational Method	Hit or Lead Compound	Experimentally Validated	Drug Discovery Stage	Ref.
Essential kinase PfPK6	Pharmacophore screening	Luceome	Yes	Lead identification	[19]
Trehalase	Homology modeling, Virtual screening, and absorption, distribution, metabolism, excretion, and toxicity (ADMET) screening	(4-(hydroxymethyl)-6-{[3,4,5-trihydroxy-6- (hydroxymethyl)oxan-2-yl]amino}-hexahydro-[1,3]dioxolo [4,5-c]pyran-2-one) Uniflorine	Yes	Lead optimization	[20]
Dihydroorotate dehydrogenase (PfDHODH), plasma membrane P-type cation translocating ATPase (PfATP4)	Structure-based pharmacophore, Density Functional Theory (DFT) study	Luteolin	Yes	Lead optimization	[21]
*P. falciparum* 3D7 and W2 strains	Deep learning, QSAR	2-(4,6-diphenyl-1,2-dihydro-1,3,5-triazin-2-yl)phenol 4-{N-[3-(morpholin-4-yl)-1,4-dioxo-1,4-dihydronaphthalen-2-yl]acetamido}benzoic acid N2-(3-fluorophenyl)-N4-[(oxolan-2-yl)methyl]quinazoline-2,4-diamine	Yes	Lead identification	[22]
*P. falciparum* 3D7 and RKL-9 strains, *P. berghei*-infected erythrocytes	Homology modeling, Reverse docking, Drug likeness screening	(1*R*,3*S*)-methyl 1-(benzo[*d*][1,3]dioxol-5-yl)-2,3,4,9-tetrahydro-1*H*-pyrido [3,4-*b*]indole-3-carboxylate	Yes	Lead optimization	[23]
*P. falciparum* apicoplast-targeted proteins	Homology modeling, molecular docking	Rifampicin	No	Drug repurposing, Lead identification	[24]
36 *P. falciparum* drug targets	Molecular docking, MD simulations	DrugBank Library	Yes	Drug repurposing, Lead identification	[25]
*P. falciparum*, strain Dd2, Enoyl-ACP (acyl carrier protein)-reductase (FabI)	QSAR	2’-substituted triclosan derivatives	No	Lead identification	[26]
p53	ADMET screening, Molecular docking	1-(1-benzyl-5-phenyl-1H-1,2,3-triazol-4-yl)-1-(4-bromophenyl)-2-((3,4-dimethylphenyl)amino)ethanol	No	Lead identification	[27]
*P. falciparum* 1-deoxy-d-xylulose-5-phosphate reductoisomerase (PfDXR)	Pharmacophore modeling, Virtual screening, Molecular docking, MD simulations	Fosmidomycin Myricetin 3-rhamnoside, 7-O-Galloyltricetiflavan (25S)-5-beta-spirostan-3-beta-ol 3-O-beta-d-glucopyranosyl-(1->2)-beta-d-glucopyranoside Oleanolic acid 28-O-beta-d-glucopyranoside	No	Lead identification	[28]
*P. falciparum* glutathione reductase	Molecular docking, MD simulations	1,2,3-triazole-linked dihydropyrimidinone quinoline hybrids	Yes	Optimization of drug structure	[29]
Falcipains	Quantum mechanics/molecular mechanics, MD simulations	N-[N-(1-hydroxycarboxyethylcarbonyl)leucylaminobutyl]guanidine (E64)	Yes	Lead optimization	[30]
	MD simulations	N-(2H-1,3-benzodioxol-5-yl)-N’-[2(1-methyl-1,2,3,4-tetrahydroquinolin-6-yl) ethyl]ethanediamide N-{3-[(biphenyl-4yl carbonyl) amino]propyl}-1H-indole-2-carboxamide	Yes	Lead optimization	[31]

**Table 2:** Binding energies of different antimalarial drugs in the two known binding sites of BSA and HSA.

Drug Name	Binding Free Energy (kJ mol^−1^) ^a^		Ref.
	Site I	Site II	
Lumefantrine	−29.83	−26.95	[102]
TO1 ^b^	−28.6	-	[103]
TO2 ^b^	−26.1	-	
Piperaquine	−40	-	[108]
M1 ^c^	−28	-	
M2 ^c^	−29	-	
M3 ^c^	−32	−33	
M4 ^c^	−41	-	
M5 ^c^	−42	−36	
Artemether	-	−26	[110]
Tri-methoxy flavone	−28.01	−32.40	[115]

^a^ Dash line indicates that the data was not reported. ^b^ Artemisinin analogues. ^c^ Piperaquine derivatives.

## Data Availability

Not applicable.
